# BB-Cl-Amidine as a novel therapeutic for canine and feline mammary cancer via activation of the endoplasmic reticulum stress pathway

**DOI:** 10.1186/s12885-018-4323-8

**Published:** 2018-04-12

**Authors:** Melissa M. Ledet, Robyn Anderson, Rebecca Harman, Aaron Muth, Paul R. Thompson, Scott A. Coonrod, Gerlinde R. Van de Walle

**Affiliations:** 1 Baker Institute for Animal Health, College of Veterinary Medicine, Cornell University, Ithaca, NY USA; 2 Department of Biochemistry and Molecular Pharmacology, University of Massachusetts Medical School, Worcester, MA USA

**Keywords:** Peptidyl arginine deiminase, Endoplasmic reticulum stress, Mammary cancer, BB-cl-Amidine

## Abstract

**Background:**

Mammary cancer is highly prevalent in dogs and cats and results in a poor prognosis due to critically lacking viable treatment options. Recent human and mouse studies have suggested that inhibiting peptidyl arginine deiminase enzymes (PAD) may be a novel breast cancer therapy. Based on the similarities between human breast cancer and mammary cancer in dogs and cats, we hypothesized that PAD inhibitors would also be an effective treatment for mammary cancer in these animals.

**Methods:**

Canine and feline mammary cancer cell lines were treated with BB-Cl-Amidine (BB-CLA) and evaluated for viability and tumorigenicity. Endoplasmic reticulum stress was tested by western blot, immunofluorescence, and quantitative reverse transcriptase polymerase chain reaction (qRT-PCR). Canine and feline mammary cancer xenograft models were created using NOD scid gamma (NSG) mice, and were treated with BB-CLA for two weeks.

**Results:**

We found that BB-CLA reduced viability and tumorigenicity of canine and feline mammary cancer cell lines in vitro. Additionally, we demonstrated that BB-CLA activates the endoplasmic reticulum stress pathway in these cells by downregulating 78 kDa Glucose-regulated Protein (GRP78), a potential target in breast cancer for molecular therapy, and upregulating the downstream target gene DNA Damage Inducible Transcript 3 (*DDIT3*). Finally, we established a mouse xenograft model of both canine and feline mammary cancer in which we preliminarily tested the effects of BB-CLA in vivo.

**Conclusion:**

We propose that our established mouse xenograft models will be useful for the study of mammary cancer in dogs and cats, and furthermore, that BB-CLA has potential as a novel therapeutic for mammary cancer in these species.

**Electronic supplementary material:**

The online version of this article (10.1186/s12885-018-4323-8) contains supplementary material, which is available to authorized users.

## Background

Mammary cancer is highly prevalent in dogs and cats, representing the most common cancer in intact bitches and the third most common neoplasm in queens [1, 2]. There are five basic classes of mammary cancer treatments in dogs and cats: surgery, radiation therapy, chemotherapy, hormone therapy and immunotherapy. Because the effectiveness of the latter four therapies is uncertain, surgery remains the treatment of choice [3]. Unfortunately, tumor recurrence after surgery is common in both species and adjuvant treatment with chemotherapeutics often fails [4–6]. Therefore, there is an urgent need to develop more effective treatments for feline and canine mammary cancer.

Recent human and mouse studies have suggested that peptidyl arginine deiminase enzymes (PAD) could represent a novel target for epigenetic cancer therapy. The PAD family is made up of 5 (PAD1–4 and PAD6) calcium-dependent enzymes that convert positively charged arginine residues to neutrally charged citrulline target proteins, such as histones, in a process called citrullination [7]. PAD are involved in cell differentiation, apoptosis, embryonic development and gene regulation, and each PAD isozyme has a unique tissue distribution pattern and substrate preference, which most likely confers biological specificity [7]. PAD2 represents the ancestral PAD family member and is abundantly represented in female reproductive tissues, including the mammary gland [8]. PAD2 activity was found to be significantly increased during human breast cancer progression and to be predictive of human breast cancer recurrence [9, 10]. Interestingly, PAD2 was recently identified in feline and canine carcinomas, suggesting that this oncogene may also play an important role in mammary cancer of small companion animals [11]. PAD4 was initially identified in hematopoietic cells, and elevated expression of PAD4 has been observed in various malignant tumors [12, 13]. More recently, PAD4 was also found to be highly expressed in tumorigenic human breast cancer cell lines that are resistant to conventional therapeutics [14]. However, the expression of PAD4 in feline or canine mammary cells has not been previously studied. A broad PAD inhibitor, Cl-Amidine, has been shown to have remarkable effects on human breast cancer in vitro and in vivo in a mouse xenograft model [10]. A more recent study by Wang et al. showed that the PAD4 inhibitor, YW3–56, triggers breast cancer cell death by inducing the endoplasmic reticulum (ER) stress response [15, 16]. Cancer cells are frequently exposed to exogenous and endogenous insults that induce ER stress. ER stress normally activates an intricate pathway with the goal of restoring homeostasis; however, if this stress is too prolonged or severe, cell death will occur [17–19].

Mammary cancer in dogs and cats is similar to human breast cancer in histology, gene expression, and metastatic pattern. Given these similarities, PAD inhibitors could potentially serve as novel therapeutics for mammary cancer in these species as well [20]. To begin exploring this possibility, we evaluated the effects of BB-Cl-Amidine (BB-CLA), a more potent derivative of Cl-Amidine [21], on normal and tumoral canine and feline mammary cells in vitro and in vivo, and studied the potential mechanisms underlying these effects.

## Methods

### Cell lines

The immortalized, non-malignant canine mammary epithelial cell line (CMEC), established in-house as previously described [20], the canine mammary carcinoma cell line REM134 [22], the immortalized, non-malignant feline mammary epithelial cell line (FMEC), a kind gift from Dr. John Parker (Baker Institute for Animal Health, Cornell University, Ithaca, NY) [23], and the feline mammary adenocarcinoma cell line K12–72.1, a kind gift from William Hardey Jr. (School of Veterinary Medicine, Cornell University, Ithaca, NY) [24], were cultured in DMEM supplemented with 10% fetal bovine serum (Atlanta Biologicals, Lawrenceville, GA) and 1% penicillin/streptomycin. All cell lines were maintained at 37 °C in a humidified environment with 5% CO_2_.

### Antibodies and chemicals

The rabbit anti-human PAD2 and PAD4 antibodies were obtained from Proteintech (12110–1-AP; Rosemont, IL) and Sigma-Aldrich (P4749; St. Louis, MO), respectively. The rabbit anti-human caspase-3, activating transcription factor 4 (ATF4), 78 kDa Glucose-regulated Protein (GRP78) and beta actin antibodies were obtained from Abcam (ab4051; Cambridge, UK), Santa Cruz Biotechnology (sc-200; Dallas, TX), LS Bio (LS-C312961; Seattle, WA), and Abcam (ab8227), respectively. The mouse anti-dog/cat estrogen receptor alpha (ERα) antibody was obtained from Thermo Fisher Scientific (MA5–13065; Waltham, MA). The human anti-modified citrulline antibody was obtained from Millipore (MABS487; Darmstadt, Germany). Alexa 488-conjugated goat anti-rabbit antibodies, HRP-conjugated goat anti-mouse antibodies, HRP-conjugated goat anti-rabbit and HRP-conjugated goat anti-human antibodies were all obtained from Jackson ImmunoResearch Labs (West Grove, PA). The PAD inhibitor, BB-CLA, was synthesized as previously described and diluted in dimethyl sulfoxide (DMSO) [25].

### Cell viability assays

After 48 h of treatment with either BB-CLA (0 to 20 μM) or DMSO (control), a 3-(4,5-dimethylthiazol-2-yl)-2,5-diphenyltetrazolium bromide (MTT) in vitro toxicology assay (Sigma-Aldrich) was carried out per manufacturer’s instructions and absorbance was measured at 570 nm on a Multiskan EX plate reader (Thermo Fisher Scientific). Optical densities of wells treated with BB-CLA were compared with those treated with equivalent amounts of DMSO to determine cell viability. Values were expressed relative to DMSO treated wells.

### Soft agar assays

Cells were detached using (0.25%) Trypsin-EDTA (Corning Life Sciences, Manassas, VA) and counted. Soft agar assays were performed as previously described [26]. To this end, 2 mL of 0.6% 2-hydroxyethylagarose melted in appropriate culture medium were pipetted into wells of 6-well culture plates and plates were held at 4 °C for 15 min until the agarose solidified. Ten thousand cells per well were gently resuspended in 1.5 mL 0.3% 2-hydroxyethylagarose melted in appropriate culture medium with either DMSO or 10 μM BB-CLA, and layered over the base agarose. Cells were cultured in soft agar for 14 days at 37 °C with 5% CO_2_. Every 3 days, cultures were fed with 1 mL 0.3% 2-hydroxyethylagarose melted in appropriate culture medium with either DMSO or BB-CLA. Cultures were photographed at 10× using a Nikon Diaphot-TMD inverted light microscope with an attached Cohu CCD camera (Nikon, Melville, NY). The number of spheres, defined as clusters of cells increasing in size due to cell division [20], in 30 fields were counted.

### Gene expression analyses

Cells were seeded at a density of 2 × 10^5^ in T25 tissue culture flasks. After 24 h, culture medium was removed, cell monolayers were rinsed with PBS, and cells were incubated in either 10 μM BB-CLA or DMSO for 3 or 6 h. Subsequently, mRNA was extracted from the cells using an RNeasy Plus Kit (QIAGEN, Valencia CA) and cDNA was synthesized using M-MLV Reverse Transcriptase (USB, Cleveland, OH), both according to manufacturer’s protocols. The amplification reactions were performed using Invitrogen reagents (Life Technologies) and an Eppendorf Mastercycler. Following standard gel electrophoresis, products were visualized using a ChemiDoc MP Imaging System (Bio-Rad). SYBER green-based quantitative reverse transcriptase polymerase chain reaction (qRT-PCR) assays were carried out on an Applied Biosytems 7500 Fast Real Time PCR instrument (Applied Biosystems, Carlsbad, CA) to determine fold changes in gene expression. The comparative Ct method was used to quantify gene expression levels where ΔΔCt = ΔCt (sample) – ΔCt (reference). The reference genes Ribosomal Protein L 32 (*RPL32*) and Ribosomal Protein L 17 (*RPL17*) were used to normalize canine and feline samples, respectively. Primers to amplify *RPL32, RPL17, PAD2, PAD4*, *ERα*, DNA Damage Inducible Transcript 3 (*DDIT3*), and DNA Damage Inducible Transcript 4 (*DDIT4*) were designed using Primer3 software, based on canine and feline sequences found in the National Center of Biotechnology Information (NCBI) GenBank. Primer sequences are listed in Table 1. All samples were run in triplicate.

**Table 1:** Primers used in the study

Species	Gene	Forward Primer (5′ → 3′)	Reverse Primer (5′ → 3′)
Canine	*RPL32*	TTGAAGTGCTGCTGATGTGC	GGGATTGGTGACTCTGATGG
Feline	*RPL17*	AAGAACACACGGGAAACTGC	CTGGGCACACCTACCAACTC
Canine	*PAD2*	AGCAAGCGGATCACCATC	ATGTCACGGTTCCAGTCCAG
Feline	*PAD2*	CAGCAAGCGAATCACCATC	AGGATGTCACGGTTCCAGTC
Canine	*PAD4*	GTCGGGAGGAAGAAGTACCC	CATCTCCTGGCTCTCCTTG
Feline	*PAD4*	AGGACGTTCTGTCCAACGAG	GCTTTAACACCTCCCGGTTC
Canine	*ERα*	AGGAAGAATGTTGAAACACAAAC	AGCAAGTTAGGAGCAAAGAAGAG
Feline	*ERα*	ATGTTGCTGGCTACGTCATCTC	GGTCCTTCTCTTCCAGAGACTTC
Canine	*DDIT3*	AGCTGGAAGCCTGGTATGAG	CAGTCAGCCAAGCCAGAGAG
Feline	*DDIT3*	AGCTGGAAGCCTGGTATGAG	CAGAGAGGCAGGGTCAAGAG
Canine	*DDIT4*	CTGGACAGCAGCAACAGTG	CATCAGGTTGGCACACAAG
Feline	*DDIT4*	CTGGACAGCAGCAACAGTG	GCACACAAGTGCTCATCCTC

### Flow cytometry analyses

Cells were collected using accutase (Innovative Cell Technologies, San Diego, CA), fixed for 15 min in 4% paraformaldehyde (PFA), permeabilized and blocked in 0.1% Triton-X with 1% normal goat serum in PBS with 1% bovine serum albumin BSA for 1 h, and stained with PAD2 or PAD4 antibodies, diluted 1:50 in PBS with 1% BSA, for 1 h. Staining with an isotype control antibody (Abcam), diluted 1:50 in PBS with 1% BSA, or no antibody stain were included as controls. Cells were washed three times in PBS and incubated with Alexa 488-conjugated secondary goat anti-rabbit antibodies diluted 1:500 in PBS with 1% BSA, for 30 min. After washing, 10,000 cells were analyzed on a Gallios flow cytometer controlled by Kaluza for Gallios software (Beckman Coulter, Indianapolis, IN). Data analysis was conducted using Kaluza Analysis software (Beckman Coulter, Indianapolis, IN).

### Western blot (WB) analyses

Cells were lysed in RIPA buffer containing a 1X general protease inhibitor. Protein concentration was determined with a BCA protein assay (Thermo Fisher Scientific) prior to gel loading to ensure loading of an equal amount of protein (40 μg). 6X reducing sample buffer was added to yield a final concentration of 1X and lysates were boiled for 10 min at 95 °C. Samples were subjected to SDS polyacrylamide gel electrophoresis on a 10% gel and transferred to Immobilin PVDF membranes (Millipore, Billerica, MA) using a transblot turbo system (Biorad, Hercules, CA). Membranes were blocked in 5% BSA diluted in Tris buffered saline (TBS) and incubated with PAD2 or PAD4 antibodies diluted 1:1000, GRP78 antibodies diluted 1:500, ERα antibodies diluted 1:200, or modified-citrulline antibodies diluted 1:1000 in TBS for 1 h room temperature (RT) on a rotating platform. Blots were washed for 50 min (10 × 5 min) with TBS-Tween, then incubated with HRP conjugated secondary antibodies for 1 h at RT. All blots were washed for 50 min (10 × 5 min) with TBS-Tween and then visualized by chemiluminescence using Clarity Western ECL (BioRad, Hercules, CA). Membranes were stripped and probed with beta actin antibodies, diluted 1:5000, as a loading control. Gels were imaged on a BioRad ChemiDoc MP system (BioRad) and band intensities were determined using Image Lab software. Intensities of the bands of interest were divided by the intensities of loading control bands to calculate relative band intensity.

### Immunofluorescence (IF)

Cells for IF were grown in 24 well culture dishes fitted with 35 mm coverslips, rinsed with PBS and fixed in 4% PFA for 10 min. Following 3 rinses with PBS, cells were permeabilized using PBS + 1% Triton-X 100 + 1% BSA for 30 min at RT. Primary antibodies against PAD2, PAD4, ATF4, all at 1:50 dilution in PBS, or Caspase-3 at 1:100 dilution, were added to the wells for 1 h at 37 °C. Wells were rinsed 3 times with PBS and Alexa 488-conjugated secondary antibodies were added to appropriate wells. After 30 min at RT, cells were washed 3 times with PBS and DAPI was added for 5 min. After a PBS wash, coverslips were removed from wells and mounted on slides using mounting medium (Dako, Carpenteria, CA). Cells were examined with a Zeiss LSM confocal microscope (Oberkochen, Germany) and images were captured with a camera controlled by ZEN imaging software. Nine images per sample, approximately 250 cells, were analyzed and DAPI-PAD co-localization was quantified using Image J Software.

### Generation of REM134 and K12.72.1 xenografts

Both REM134 and K12.72.1 xenograft tumors were generated by injecting 1 × 10^6^ cells in 0.1 mL Matrigel (1:1) (BD Biosciences, San Jose, CA, USA) subcutaneously near the nipple of gland #4 in 6-week old female NOD scid gamma (NSG) mice (Taconic, Germantown, NY, USA), as previously reported for mammary xenograft models [10]. After two weeks, intraperitoneal injections of BB-CLA (1 mg/kg/day) or vehicle control (PBS) were initiated and carried out for 14 days. Tumor diameter was measured daily by digital caliper and the volume (mm^3^) was calculated using the formula: a^2^ x b/2, where “a” is the shortest diameter and “b” is the longest diameter of the tumor. Results are reported as mean ± SD. After 14 days, tumors were removed and added to 10% buffered formalin. Parrafin sections were stained with hematoxylin and eosin to determine for mitotic rate and necrosis. A rodent pathologist estimated the percent of necrosis by looking at the entire tumor at low power. For mitotic rate, the number of mitotic figures were counted in the area with the highest mitotic rate in a 400× field. To measure apoptosis, a Terminal deoxynucleotidyl transferase (TdT) dUTP Nick-End Labeling (TUNEL) assay (Sigma-Aldrich) was used according to manufacturer’s instructions and images were quantified using Image J Software. Three mice per group were used for each treatment. All mouse experiments were reviewed and approved by the Institutional Animal Care and Use Committees (IACUC) at Cornell University (#2013–0022).

### Statistical analyses

All experiments were repeated at least three times and results were expressed as the mean ± STDEV from three independent experiments. Data were analyzed by the Student’s T-test and MTT assays were analyzed by one-way ANOVA and *p* values < 0.05 were considered statistically significant. * indicates *P* < .05, ** *P* < .01, *** *P* < .001, and **** *P* < .0001.

## Results

### BB-CLA selectively inhibits growth of canine and feline mammary tumor cells compared to normal mammary epithelial cells

**Fig. 1:**
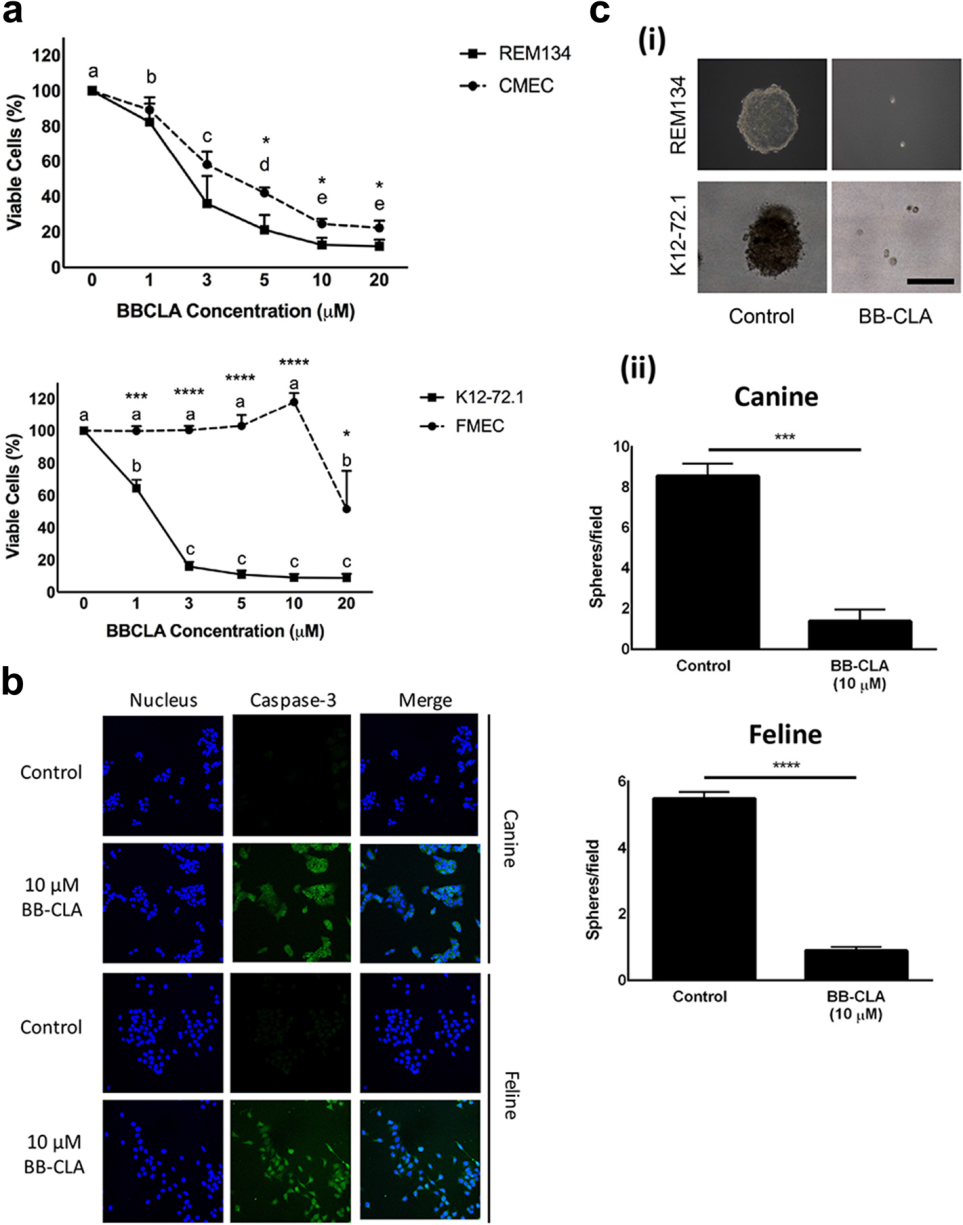
Effect of BB-CLA on viability and tumorigenicity of canine and feline mammary cancer cell lines. **a** Cells were treated for 48 h with different concentrations of BB-CLA and viability was evaluated using MTT assays. Values are expressed relative to cells treated with DMSO (control). Letters indicate significant differences between doses for each cell line (one-way ANOVA) and asterisks indicate significant differences between cell lines (T-test). **b** Cells were treated for 6 h with 10 μM BB-CLA and probed with antibodies to Caspase-3. Representative images are shown. **c** Soft agar assays were established in which cells were treated with 10 μM BB-CLA or DMSO (control). Representative images after two weeks of treatment (i) and quantification of spheres (ii) are shown. Scale bar: 10 μm. *P < 0.05, ***P < 0.001, ****P < 0.0001, *n* = 3. Data are presented as mean ± standard deviation

To start exploring the potential of peptidyl arginine deiminase enzyme (PAD) inhibitors as anti-cancer drugs to treat canine and feline mammary cancer, the tumoral cell lines REM134 (canine) and K12–72.1 (feline) were incubated with increasing doses of BB-Cl-Amidine (BB-CLA) for 48 h and evaluated for viability using MTT assays. A dose-dependent decrease in cell viability was observed in both tumoral cell lines and reached significance starting at 1 μM for both REM134 and K12–72.1 (Fig. 1a). Importantly, this effect on cell viability was lower in non-malignant canine (CMEC) and feline (FMEC) mammary cells, and for FMEC, only became statistically different at the higher doses of BB-CLA, indicating that this drug preferentially affects tumoral cells (Fig. 1a). A significant difference in effect between non-malignant and tumoral cells was observed beginning at 1 μM for feline cells and 5 μM for canine cells (Fig. 1a). We used 10 μM BB-CLA for all future experiments, based on the significant effect on viability of the tumoral cells from both species. To follow up on the underlying mechanism for the reduced viability after BB-CLA treatment, cells were stained for caspase-3 at 6 h post BB-CLA treatment. In contrast to non-treated REM134 and K12–72.1, BB-CLA-treated cells stained positive for caspase-3, indicating that the cells underwent apoptosis in response to BB-CLA (Fig. 1b). We then evaluated the effect of BB-CLA on tumorigenicity by using soft agar assays, which are established to be good predictors of in vivo tumorigenicity [10]. An equivalent amount of DMSO was added to a separate set of wells as a control. Compared to the control cells, REM134 and K12–72.1 treated with BB-CLA formed significantly fewer spheres and these spheres were significantly smaller (Fig. 1c).

### Normal and tumoral feline and canine mammary cells express similar levels of PAD2 and PAD4

**Fig. 2:**
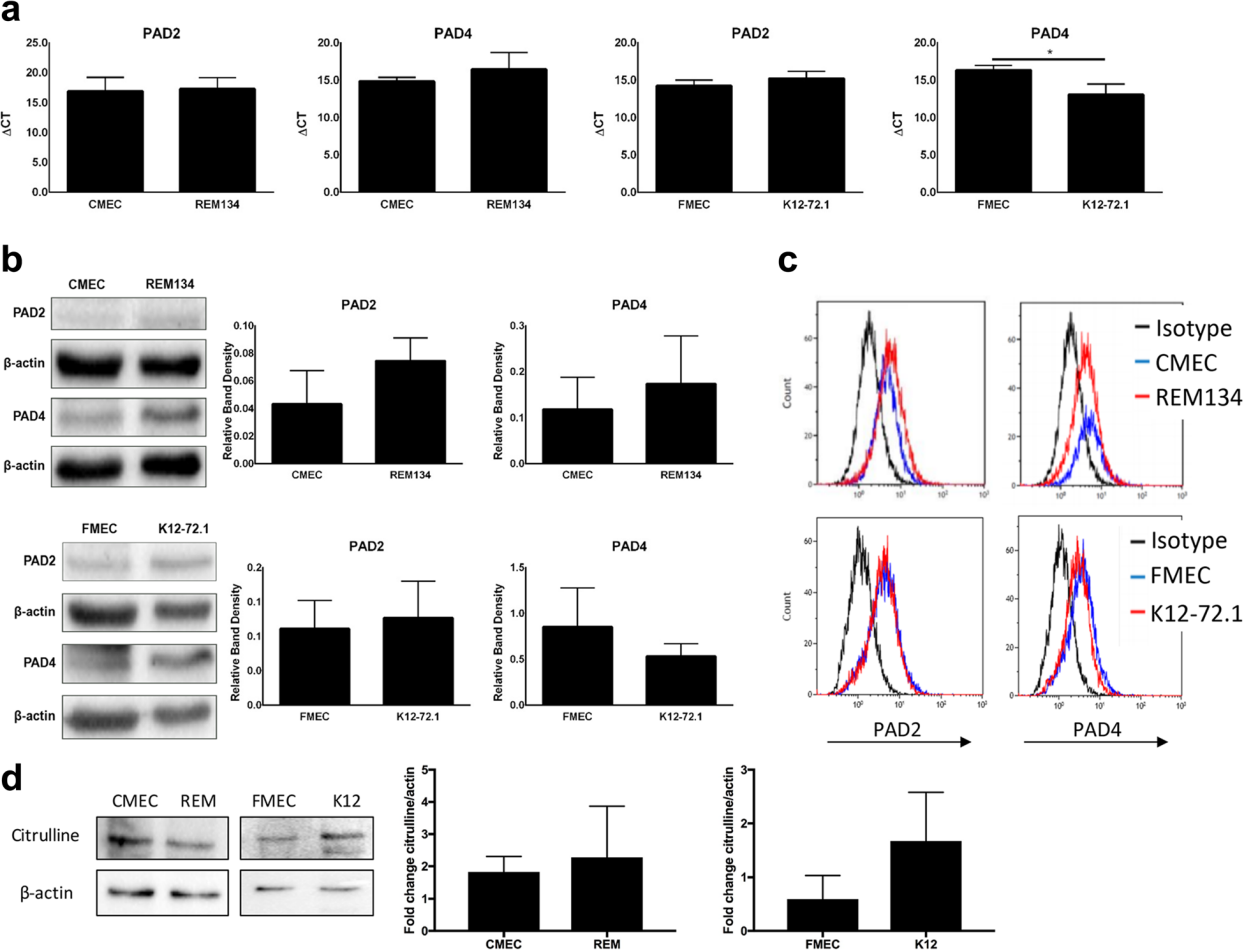
Expression of PAD2 and PAD4 in non-malignant and tumoral canine and feline mammary cell lines. **a** Expression levels of the genes PAD2 and PAD4 in canine and feline normal and tumoral mammary cell lines as determined by qRT-PCR. **b** Protein expression in whole cell lysates subjected to SDS-PAGE and immunoblot analyses probed with PAD2 or PAD4 antibodies. β-actin was included as a loading control. Representative Western blots and quantifications are shown. Quantification is represented as the fold change of PAD2/4 band density over β-actin band density. **c** Protein expression in cells probed with either PAD2 or PAD4 antibodies and subjected to flow cytometry analyses. **d** Protein expression in whole cell lysates subjected to SDS-PAGE and immunoblot analyses probed with anti-modified citrulline antibodies. β-actin was included as a loading control. Representative Western blots and quantifications are shown. Quantification is represented as the fold change of modified-citrulline band density over β-actin band density. *P < 0.05, *n* = 3. Data are presented as mean ± standard deviation

**Fig. 3:**
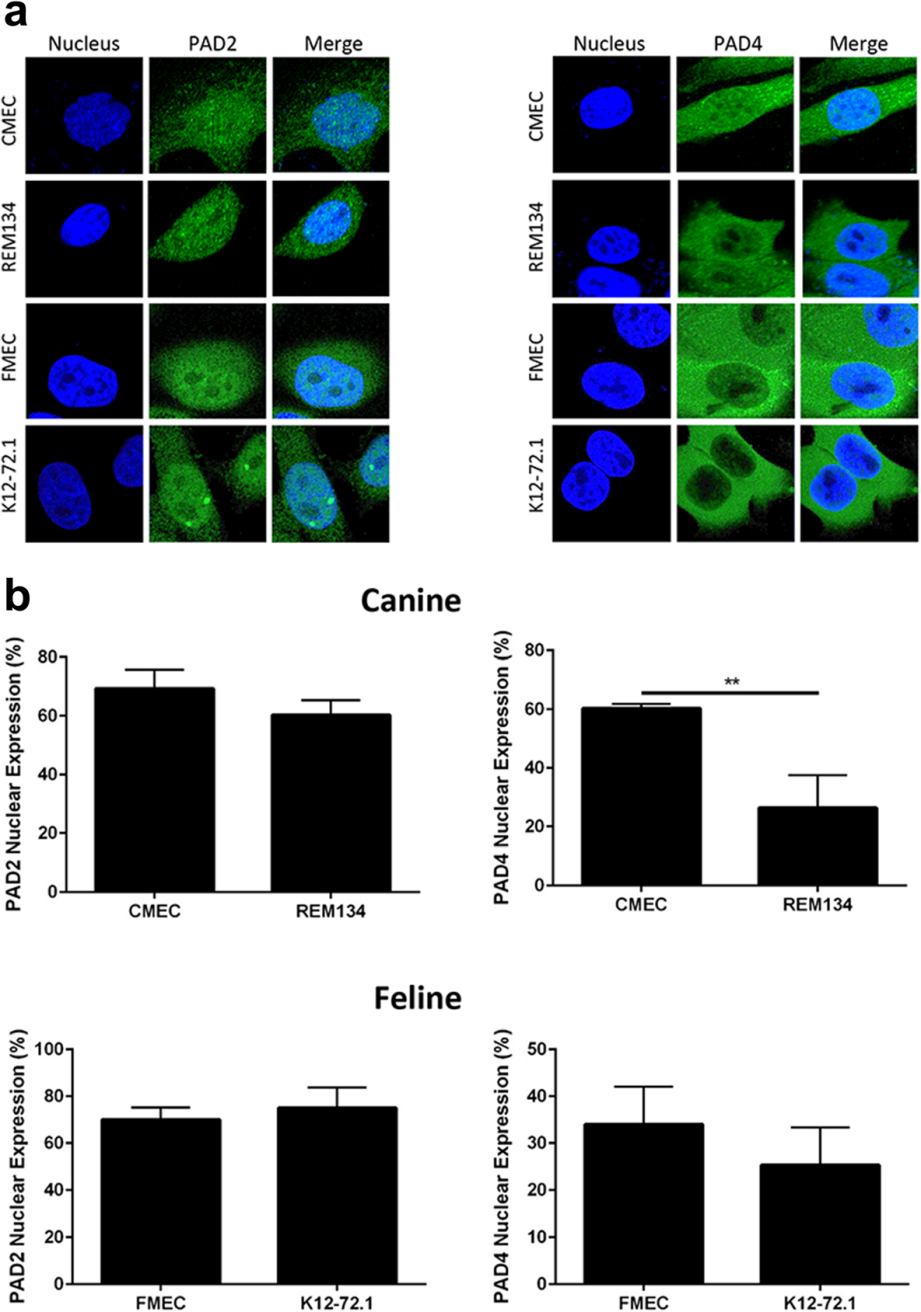
Cellular localization of PAD2 and PAD4 in non-malignant and tumoral canine and feline mammary cell lines. Protein localization in cells probed with PAD2 or PAD4 antibodies and subjected to immunofluorescence. Representative fluorescence pictures (**a**) and quantifications, using Image J Software, (**b**) are shown. **P < 0.01, *n* = 3. Data are presented as mean ± standard deviation

To date, no data are available on the expression of PAD enzymes in canine or feline mammary cell lines. Therefore, we decided to evaluate PAD expression in both normal and tumoral canine and feline cell lines, to see whether a differential expression could be observed similar to what has been described for the human MCF10AT mammary cell line series, where PAD2 was more abundantly expressed in the tumoral cell lines compared to the normal MCF10a cell line [9, 11]. We looked at PAD2 and PAD4 specifically, since these enzymes have been described to be expressed in the mammary gland [11]. First, we used quantitative (q) RT-PCR to compare mRNA expression levels of these two PAD enzymes in the different cell lines, and in contrast to the human results, PAD2 mRNA expression levels were virtually indistinguishable between normal and tumoral cells of both canine and feline origin (Fig. 2a). PAD4 mRNA levels were also similar between normal and tumoral canine mammary cells and a slight, but significant, increased expression was observed in the feline tumoral cell line K12–72.1 compared to normal FMEC, as indicated by the lower ΔCT values (Fig. 2a). Next, we evaluated PAD2 and PAD4 expression on a protein level, since the level of translation of PAD2 and PAD4 could be different between normal and tumoral cells. Western Blot (WB) analyses showed that PAD2 and PAD4 proteins were expressed similarly in normal and tumoral cells of both species (Fig. 2b). Flow cytometry analyses corroborated these findings (Fig. 2c), and combined with the qRT-PCR results, led us to conclude that PAD2 and PAD4 are expressed at similar levels in normal and tumoral canine and feline mammary cell lines. Finally, we determined the relative amount of citrulline in the cells as a measure of PAD activity using a modified anti-citrulline antibody, as previously described [27, 28]. No difference was detected by western blot (Fig. 2d), further supporting that the levels of PAD enzymes are similar in these canine and feline non-malignant and tumoral cell lines. Since immunohistochemistry studies previously showed a decrease in PAD2 nuclear staining in canine and feline mammary carcinoma tissues compared to normal mammary epithelial tissues [26, 29], we decided to evaluate the cellular localization of PAD2 and PAD4 in our cell lines using immunofluorescence (IF). We found that PAD2 was primarily located in the nuclei of the cells, irrespective of species or disease status (normal versus tumoral) (Fig. 3a & b). PAD4 was expressed in both nuclei and cytoplasm, but PAD4 nuclear expression was lower in tumoral cells compared to normal mammary cells in both species, which reached significance in canine, but not feline cells (Fig. 3a & b).

### BB-CLA activates the endoplasmatic reticulum (ER) stress pathway in canine and feline tumoral mammary cells

**Fig. 4:**
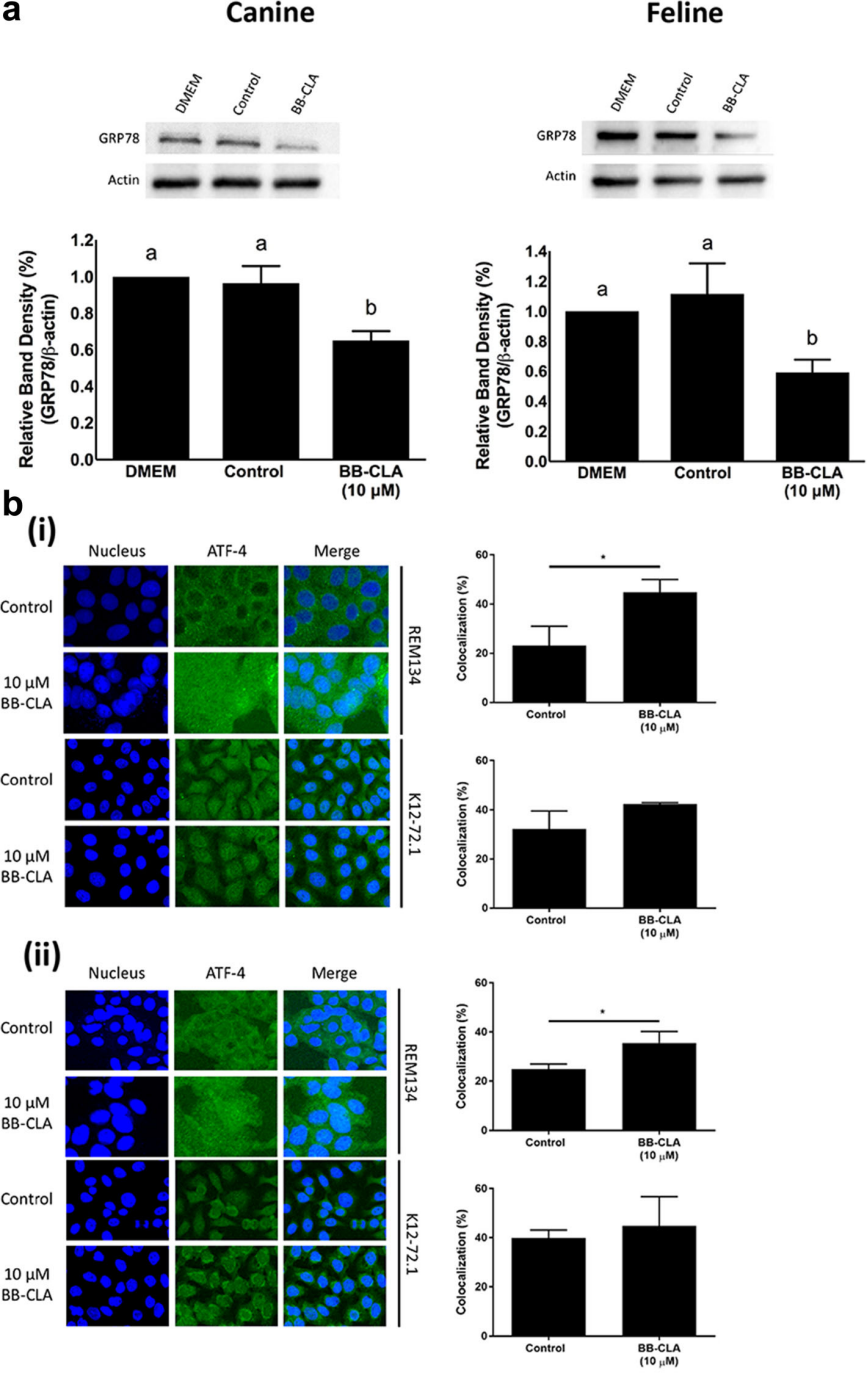
Endoplasmic reticulum (ER) stress activation in canine and feline tumoral mammary cell lines after treatment with BB-CLA. **a** Whole cell lysates treated for 3 h with DMEM (untreated), DMSO (control), or 10 μM BB-CLA were subjected to SDS-PAGE and immunoblot analyses probed with 78 kDa glucose-regulated protein (GRP78). β-actin was included as a loading control. Representative Western blots and quantifications are shown. Quantification is represented as the fold change of GRP78 band density over β-actin band density. **b** Cells were treated with BB-CLA for 3 h (i) and 6 h (ii), probed with ATF4 antibodies and subjected to immunofluorescence. Representative fluorescence pictures and quantifications, using Image J Software, are shown. *P < 0.05, *n* = 3. Data are presented as mean ± standard deviation

**Fig. 5:**
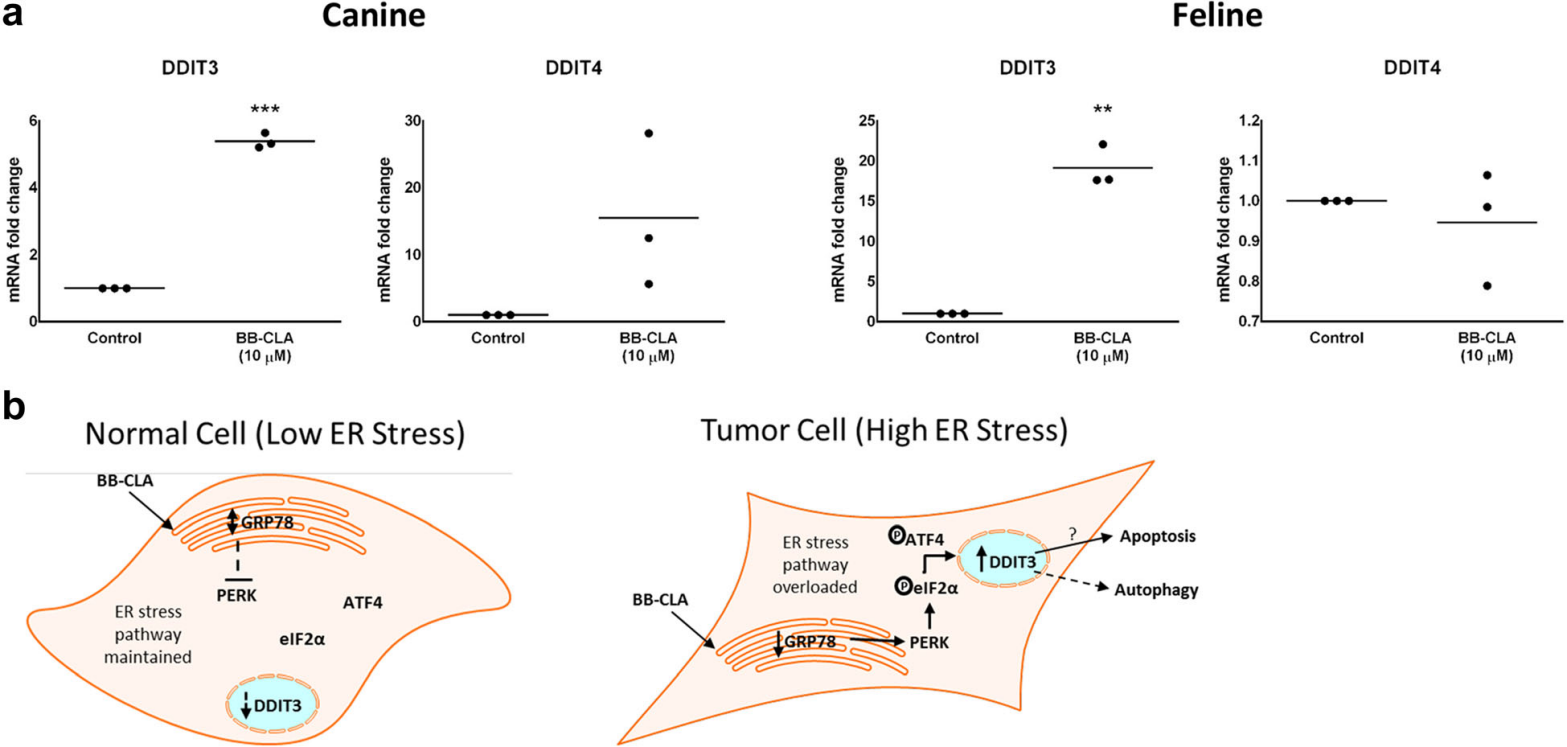
*DDIT3* gene expression indicates endoplasmic reticulum (ER) stress activation after treatment with BB-CLA. **a** Expression levels of *DDIT3* and *DDIT4* mRNA in canine and feline and tumoral mammary cell lines after 6 h of BB-CLA or DMSO (control) treatment as determined by qRT-PCR. **P < 0.01, ***P < 0.001. **b** Schematic representation of the findings presented in this study. In normal cells, baseline ER stress is low, thus any stress as a result of BB-CLA treatment is managed by the pathway at low concentrations. In tumor cells, where baseline ER stress is already high, any additional stress from BB-CLA treatment overloads the pathway leading to the initiation of cell death pathways. *n* = 3. Data are presented as mean ± standard deviation

Our data showing no differential expression of both PAD2 and PAD4 between normal and tumoral mammary epithelial cells seems to indicate that PAD inhibition is not the primary mechanism for the observed BB-CLA-induced cell death and reduced tumorigenicity of the tumoral cells, as previously reported [16]. Indeed, no changes in citrullination were observed 6 h post treatment (Additional file 1: Figure S1), although upstream inhibition of PAD activity cannot be ruled out completely. So we decided to explore whether endoplasmic reticulum (ER) stress activation could be the mode of action of BB-CLA in our canine and feline tumoral cells, based on a recent study where the activation of this pathway was demonstrated in estrogen receptor (ERα)-negative breast cancer cells using another PAD inhibitor [19, 30]. Before evaluating the ER stress activation pathway, we first confirmed the ERα negative status of our different cell lines, both at the mRNA and protein level (Additional file 2: Figure S2A & B). To study the ER stress pathway in more detail, three important checkpoints in this pathway were evaluated: (i) expression of 78 kDa Glucose-regulated Protein (GRP78), a chaperone protein in the unfolded protein response pathway [16], (ii) localization of Activating Transcription Factor 4 (ATF4), a transcriptional activator that regulates induction of proteins in response to ER stress [31], and (iii) expression of the DNA Damage Inducible Transcript 3/4 (*DDIT3/4*) genes, which are multifunctional transcriptions factors induced by ATF4 [32, 33]. GRP78 is upregulated in a variety of cancer cell lines and is elevated in primary breast tumors compared with benign mammary tissue [19, 30]. After ER stress, GRP78 is titrated away which releases its hold on the ER-transmembrane signaling molecules and activates the unfolded protein response pathway [16]. Using WB analyses, we could detect a significant decrease in GRP78 expression in both canine and feline tumoral cells in the presence of BB-CLA at 3 h post treatment, indicating that BB-CLA activates the early steps in the ER stress pathway and may represent a novel therapeutic for targeting GRP78 (Fig. 4a). Concurrently, IF staining for ATF4 showed a significant increase in nuclear localization of ATF4 in canine tumoral cells at 3 h and 6 h post BB-CLA treatment (Fig. 4b(i) & (ii), respectively). In contrast, nuclear ATF4 expression was already present in feline tumoral cells, even before BB-CLA treatment, and did not significantly increase at 3 h or 6 h post BB-CLA treatment (Fig. 4b(i) & (ii), respectively). Since translocalization of ATF4 into the nucleus results in transcription of several downstream genes such as *DDIT3* and *DDIT4* [34–36], we decided to determine the expression of these genes after BB-CLA treatment using qRT-PCR. We found a significantly increased expression of *DDIT3*, but not *DDIT4*, in both canine and feline tumoral cells at 6 h post BB-CLA when compared to DMSO-treated, control tumoral cells (Fig. 5a). This increase in *DDIT* expression was not seen in the non-malignant mammary cells of both species, i.e. FMEC or CMEC, treated with BB-CLA, indicating that this was a tumor cell-specific response (data not shown). Collectively, these data indicate that BB-CLA-induced cell death in the canine and feline mammary cancer cell lines REM134 and K12.72.1, respectively, occurs in part via the activation of the ER stress pathway and could explain the difference in susceptibility to BB-CLA between normal and tumoral cells (Fig. 5b).

### Generation of canine and feline mammary cancer xenograft mice

**Fig. 6:**
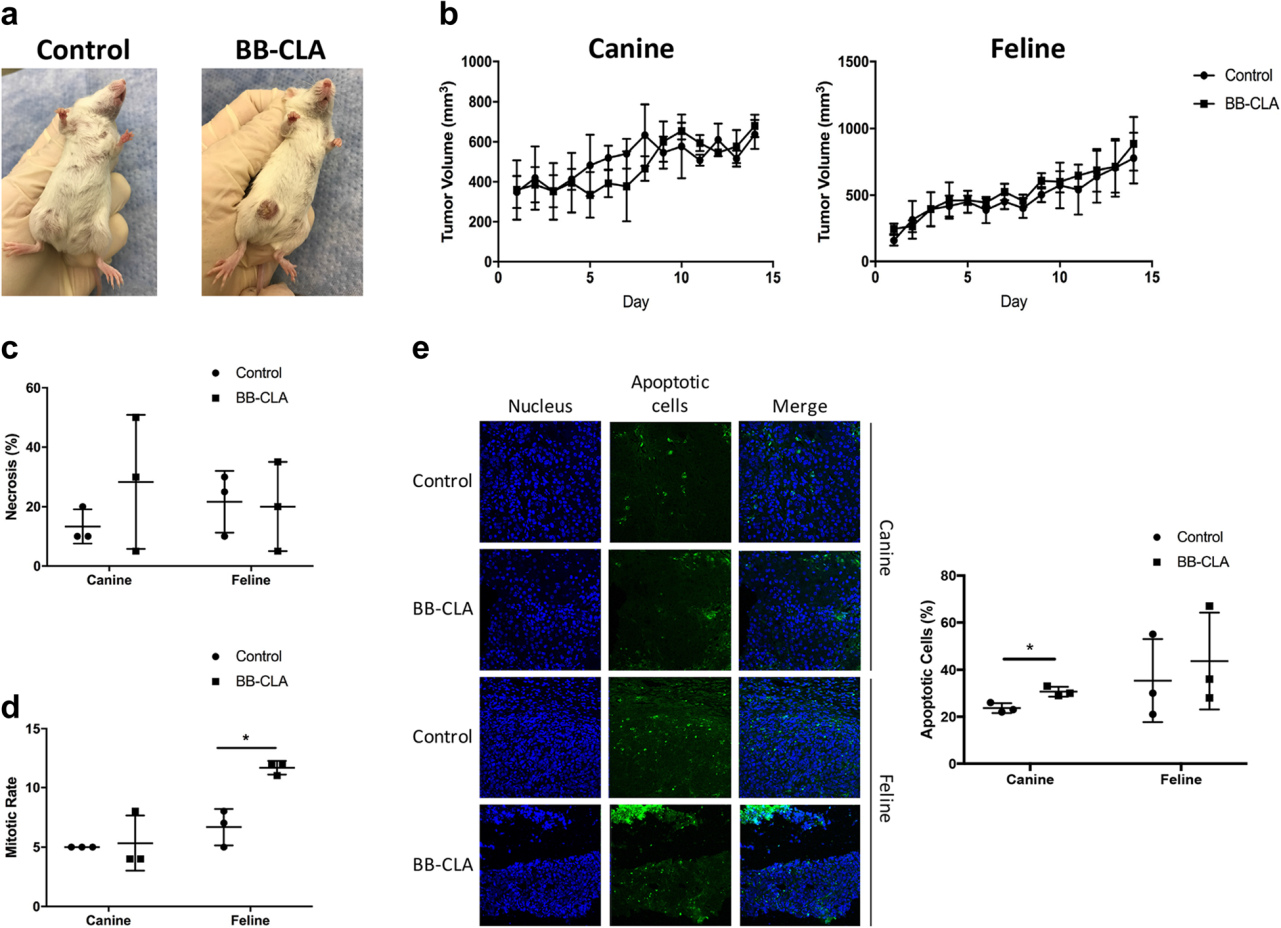
Treatment of REM134 and K12.72.1 murine xenografts. **a** Representative macroscopic pictures of tumor-bearing NGS mice at day 14 of treatment with 1 mg/kg/day BB-CLA or DMSO (control). **b** Tumor size in mice treated with BB-CLA or control over the 14-day period. Histological analyses showing necrosis percentage (**c)** and mitotic rates **(d)** at day 14 of treatment. **e** Detection of apoptosis in histological sections by Terminal deoxynucleotidyl transferase (TdT) dUTP Nick-End Labeling (TUNEL) assay, with quantification using Image J Software. *P < 0.05, *n* = 3. Data are presented as mean ± standard deviation

To test the efficacy of BB-CLA in vivo on tumors derived from the same cell lines used in our in vitro experiments, we created a mammary cancer xenograft model of each species using the REM134 (canine) and K12.72.1 (feline) cell lines. Although several canine, and one recent feline, mammary tumor xenograft models have been developed previously [37], these cell lines have not been used in an orthotopic xenograft, so we needed to validate the model with our cell lines first. To this end, mice were injected with cells in a 1:1 ratio with Matrigel in the 4th mammary gland and reproducibly developed a prominent mammary tumor within two and four weeks for REM134 and K12.72.1, respectively. These were all squamous cell carcinomas that appeared to be derived from mammary duct epithelium, and in some tumors, remnant ducts surrounded by myoepithelial cells and lined by neoplastic cells, were evident. The average tumor volume ranged around 353.45 ± 106.96 mm^3^ and 204.02 ± 60.13 mm^3^ for REM134 and K12.72.1, respectively. To preliminary evaluate the efficacy of BB-CLA in these xenograft models, the drug was injected intraperitoneally for two weeks at 1 μg/ml, as this dose was previously described to be nontoxic in mice [10]. During the treatment period, BB-CLA-treated mice were observed for changes in tumor appearance compared to the control mice in both xenograft models, and it was found that BB-CLA-treated tumors became crusty and the surrounding skin showed hair loss (Fig. 6a). Despite these striking visible changes, no difference in volume was observed during the two-week injection period (Fig. 6b). Histological examination yielded no difference in necrosis between control and treated tumors (Fig. 6c) and a slight difference in mitotic rate in the feline xenograft tumors (Fig. 6d). There was an increase in the percentage of apoptotic cells in the BB-CLA-treated canine xenograft tumors compared to the controls (Fig. 6e), similar to our in vitro data where apoptotic cells were observed in the BB-CLA-treated, but not untreated, cells (Fig. 1b). There was a slight increase in apoptotic cells in the BB-CLA-treated feline xenograft tumors also, however, this did not reach significance (Fig. 6e).

## Discussion

This study was initiated to evaluate the efficacy of the peptidyl arginine deiminase (PAD) inhibitor BB-Cl-Amidine (BB-CLA) on canine and feline mammary cancer cell lines in vitro and in vivo. Our salient findings were that BB-CLA reduces the viability and tumorigenicity of canine and feline mammary cancer cell lines at low concentrations, via the activation of the endoplasmic reticulum (ER) stress pathway, with no to minimal effects on the viability of normal mammary cells. This study also provided the first description of PAD4, and a more in-depth description of PAD2, expression in normal and tumoral mammary cells from canine and feline origin. In addition, we report on the generation of xenograft models using the cell lines REM134 and K12.72.1, and the preliminary evaluation of BB-CLA efficacy in these models.

In contrast to what has been described previously for human breast cell lines, we found a similar PAD2 and PAD4 expression between normal and tumoral mammary canine and feline cell lines. One explanation for this discrepancy between species is based on the source of the cells that were used. McElwee et al. found higher PAD2 expression in human breast cancer cells compared to normal mammary epithelial cells using the MCF10AT mammary cell line series in which the tumor cells were developed from the transformed normal mammary MCF10a cell line, and thus all cells have the same cell of origin [10, 38, 39]. In our study, the normal and tumoral feline and canine cell lines being compared were developed from different individuals for both species. Therefore, inter-animal variations in PAD2 and PAD4 levels could explain why no differences were detected between normal and tumoral cells in the present study. It would be interesting in future work to look at PAD2 and PAD4 expression in primary tumor and healthy tissue samples instead of cell lines and/or to use cell line series originating from the same animal, similar to the MCF10AT cell lines series, to determine if there are indeed inter-animal variations. Another explanation for the observed discrepancy could be the receptor status of the cell lines used. Previous data on PAD2 and PAD4 expression in human breast cells were primarily generated using estrogen receptor alpha (ERα)-positive breast cancer cells [10], whereas the canine and feline cell lines used in this study were ERα negative. This explanation is further supported by previous studies showing that PAD2 is more abundantly expressed in the luminal breast cancer subtype, which is ERα positive [16]. Additional studies should evaluate PAD expression in ERα-positive feline and canine mammary cancer cells to determine if ERα expression is indeed correlated with PAD expression.

Our results showed that treatment with the PAD inhibitor BB-CLA in canine and feline mammary cancer cells resulted in an activation of the ER stress pathway. This is in line with a recent study from Wang et al., showing that YW3–56, another PAD inhibitor, initiated the ER stress pathway through activation of ATF4 in human breast cancer cells, leading to autophagy in these cells [1, 2]. When evaluating the different genes and proteins involved in the ER stress pathway in our canine and feline cell lines, some interesting observations were made. First, we observed a difference in ATF4 localization between feline and canine mammary cancer cells, irrespective of BB-CLA treatment, and so we like to postulate that these inherent differences may be reflective of the tumoral malignancy in vivo. It is known that feline mammary cancer is on average more aggressive than canine mammary cancer, with 80% of mammary tumors being malignant in cats versus 50% being malignant in dogs, and consequently, that mammary cancer in cats has a worse prognosis [40, 41]. Also, we observed that the feline tumoral cells were more responsive to BB-CLA compared to canine tumoral cells and in parallel, we found that untreated feline cells have inherently more nuclear ATF4 and thus are at a higher baseline ER stress level. Since BB-CLA stimulates the ER stress pathway, this could explain why a greater effect was seen with this drug in the feline cells. Second, we observed a significant upregulation of the gene *DDIT3*, but not *DDIT4*, after BB-CLA treatment in both feline and canine mammary cancer cell lines, which could indicate that BB-CLA is promoting activation of the apoptosis pathway rather than the autophagy pathway in response to ER stress [18, 42–44]. Indeed, cells were positive for Caspase-3, a marker of apoptosis, after treatment with BB-CLA, indicating this is likely the predominant method for cell death after ER stress in these cells.

Despite the use of REM134 and K12.72.1 cell lines in canine and feline mammary cancer research, there were no reports of mouse orthotopic xenografts utilizing these cell lines prior to this study. REM134 has previously been used in xenografts after subcutaneous inoculation [22, 45], however orthotopic xenografts offer the advantage of a tumor microenvironment that leads to the development of tumor cells more biologically similar to clinical cases and with metastatic potential [46–48]. We found that tumors can be induced in the mammary gland reproducibly in NOD scid gamma (NSG) mice using these cell lines. Unfortunately, when we preliminary analyzed the efficacy of BB-CLA in these xenograft models, we did not find robust differences in tumor size, necrosis or mitotic rates in treated versus untreated animals. A potential explanation could be the dosage of BB-CLA used for treatment, which was based on a previous in vivo study where BB-CLA was used in a murine arthritis model [49]. It is plausible that the dosage needed for effective treatment of mammary cancer may be higher than the dose needed for the treatment of arthritis. Additionally, a longer dosage period or more frequent doses may have increased effectiveness. Future experiments in xenograft models of mammary cancer are needed to define the optimal effective BB-CLA dose. Unexpectedly, there was an increase in mitotic rate following treatment with BB-CLA in the K12–72.1 xenograft model. This would normally be associated with a poorer prognosis, however, the tumor volumes were similar in both groups. BB-CLA may have had additional effects on the tumor microenvironment that allowed for increased aggressiveness of the tumor cells, but this is unlikely as the mitotic rate was only increased in the K12–72.1 tumors and not REM134 tumors. Lastly, this finding could also be a product of low sample numbers, which should be addressed in future studies. We did, however, observe that the tumors in the BB-CLA-treated mice were crusty and that the skin showed local hair loss. A potential explanation for this observation is that other PAD, like PAD1, PAD2, and PAD3, are expressed in the skin of humans and rodents [8], and could have been affected by the PAD-inhibitor BB-CLA.

## Conclusions

Taken together, our in vitro study provides the first data on the potential of BB-CLA as a novel therapeutic to treat mammary cancer in dogs and cats. This is an exciting finding given the lack of available treatments for mammary cancer in these species. Canine and feline mammary cancer cell lines were successfully xenografted in the mouse mammary gland and will be beneficial in future research aimed at the development of mammary cancer therapeutics.

## Additional files

Additional file 1: **Figure S1**. BB-CLA does not affect citrullination in tumoral canine and feline mammary cancer cell lines. Protein expression in whole cell lysates after 6 h of 10 μM BB-CLA treatment subjected to SDS-PAGE and immunoblot analyses probed with anti-modified citrulline antibodies. β-actin was included as a loading control. Representative Western blots and quantifications are shown. Quantification is represented as the fold change of modified-citrulline band density over β-actin band density. *n* = 3. Data are presented as mean ± standard deviation. (TIFF 204 kb)

Additional file 2: **Figure S2**. Estrogen receptor expression in non-malignant and tumoral canine and feline mammary cell lines. **A.** Expression of estrogen receptor alpha (ERα) mRNA in canine and feline normal and tumoral mammary cell lines, as determined by RT-PCR. Equal cDNA loading was determined by Ribosomal Protein L (RPL) 32 (canine) and RPL17 (feline). Uterine tissue from each species was included as a positive control. **B.** ERα protein expression in whole cell lysates subjected to SDS-PAGE and immunoblot analyses probed with ERα antibodies. β-actin was included as a loading control. Canine mammary tissue was used as a positive control (+ Cont.). n = 3. Data are presented as mean ± standard deviation. (TIFF 336 kb)

## Abbreviations

ATF-4: Activating transcription factor 4
BB-CLA: BB-Cl-Amidine
CMEC: Canine mammary epithelial cells
DDIT3: DNA Damage Inducible Transcript 3
DDIT4: DNA Damage Inducible Transcript 4
DMSO: Dimethyl sulfoxide
ER: Endoplamisc reticulum
ERα: Estrogen Receptor alpha
FMEC: Feline mammary epithelial cells
GRP78: 78 kDa Glucose-regulated Protein
IF: Immunofluroescence
MTT: 3-(4,5-dimethylthiazol-2-yl)-2,5-diphenyltetrazolium bromide)
PAD: Peptyidyl Arginine Deiminase enzymes
qRT-PCR: Quantitative reverse transcriptase polymerase chain reaction
RPL17: Ribosomal protein 17
RPL32: Ribosomal protein 32
TUNEL: Terminal deoxynucleotidyl transferase (TdT) dUTP Nick-End Labeling
