# Supplementary figures and images for: BB-Cl-Amidine as a novel therapeutic for canine and feline mammary cancer via activation of the endoplasmic reticulum stress pathway
Source: BMC Cancer. 2018 Apr 12;18:412. doi: 10.1186/s12885-018-4323-8 (PMC5898062; doi:10.1186/s12885-018-4323-8)

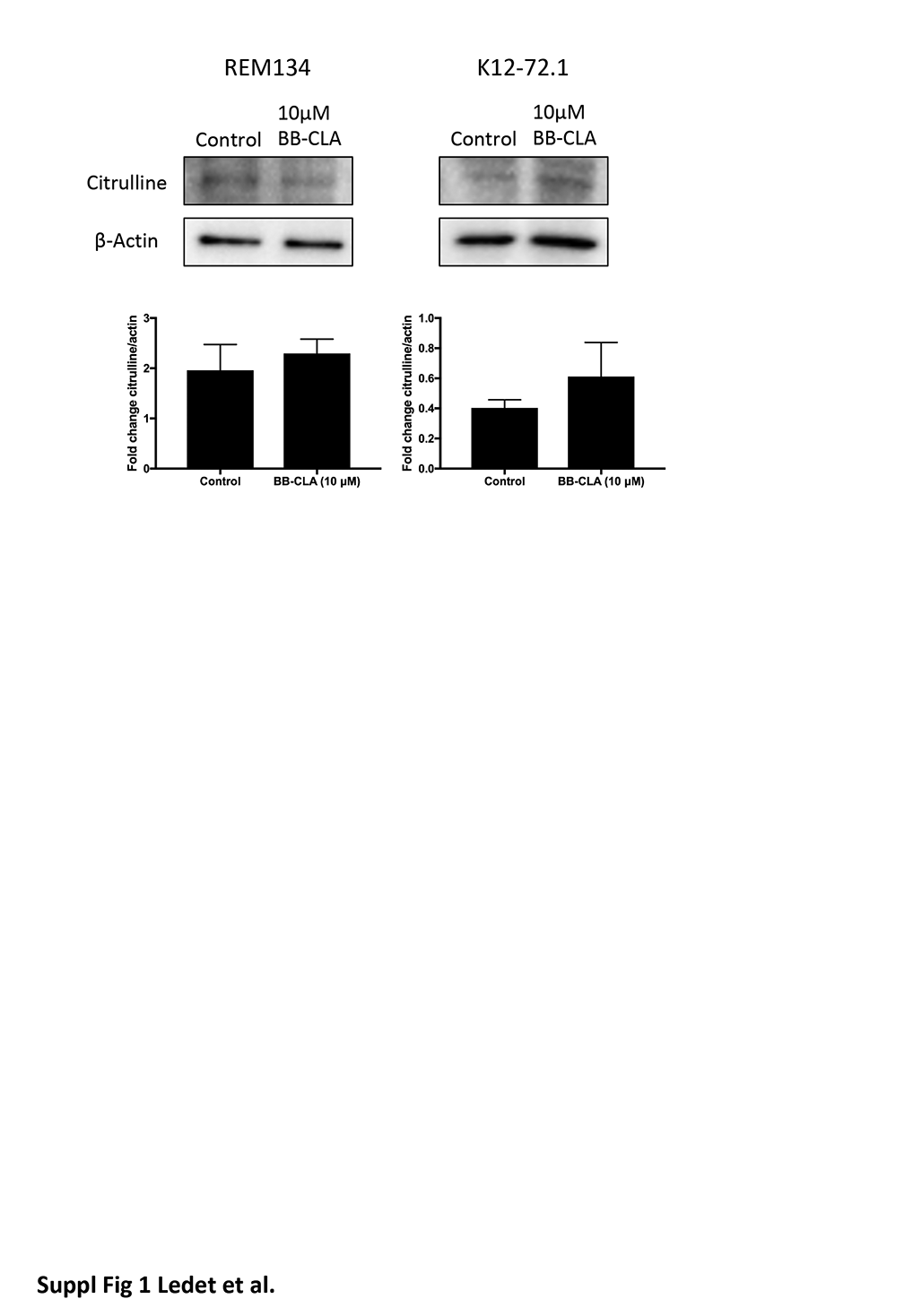

Supplement: Supplementary file 1 — Figure S1. BB-CLA does not affect citrullination in tumoral canine and feline mammary cancer cell lines. Protein expression in whole cell lysates after 6 h of 10 μM BB-CLA treatment subjected to SDS-PAGE and immunoblot analyses probed with anti-modified citrulline antibodies. β-actin was included as a loading control. Representative Western blots and quantifications are shown. Quantification is represented as the fold change of modified-citrulline band density over β-actin band density. n = 3. Data are presented as mean ± standard deviation. (TIFF 204 kb) [file 12885_2018_4323_MOESM1_ESM.tif]

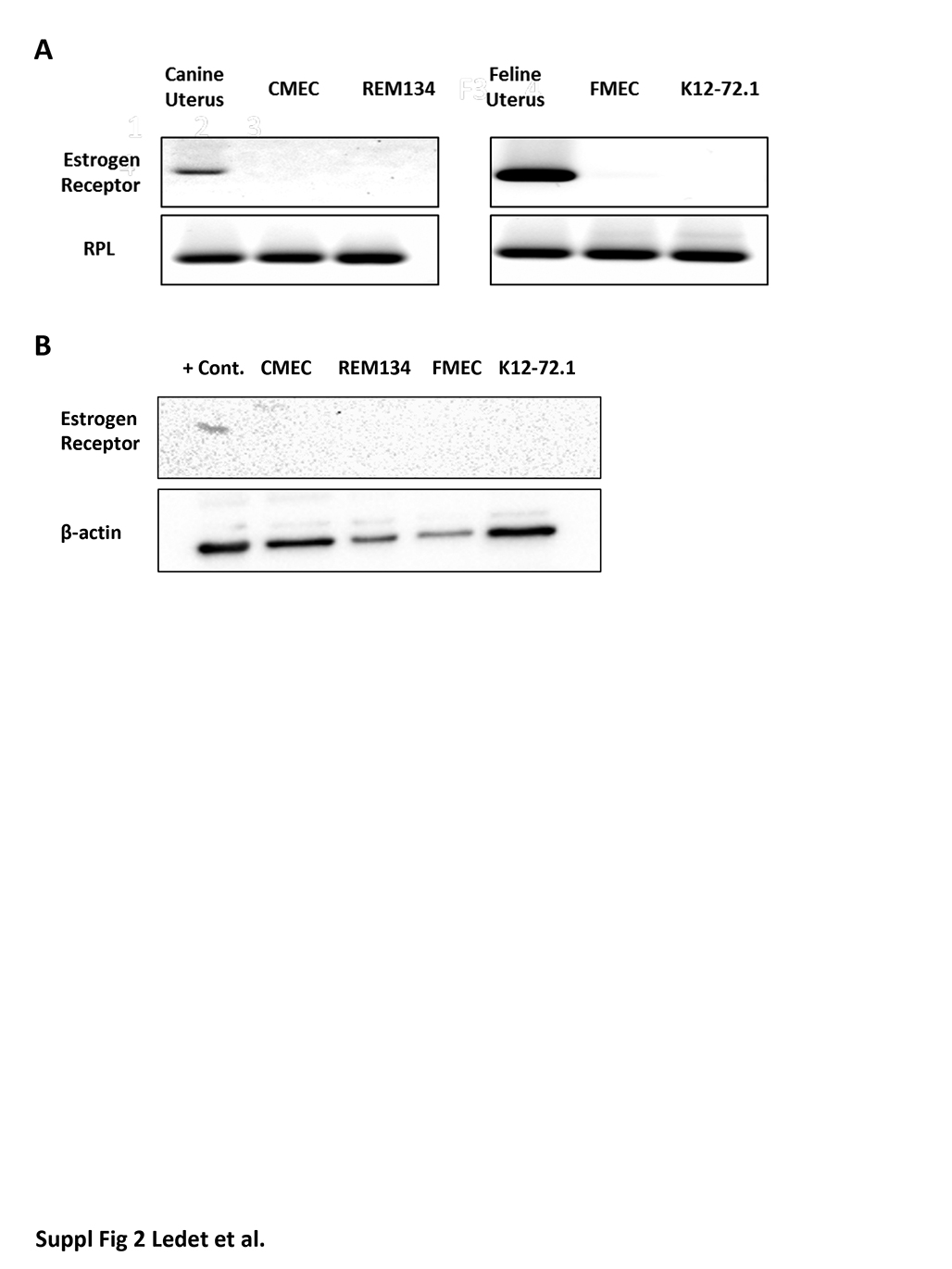

Supplement: Supplementary file 2 — Figure S2. Estrogen receptor expression in non-malignant and tumoral canine and feline mammary cell lines. A. Expression of estrogen receptor alpha (ERα) mRNA in canine and feline normal and tumoral mammary cell lines, as determined by RT-PCR. Equal cDNA loading was determined by Ribosomal Protein L (RPL) 32 (canine) and RPL17 (feline). Uterine tissue from each species was included as a positive control. B. ERα protein expression in whole cell lysates subjected to SDS-PAGE and immunoblot analyses probed with ERα antibodies. β-actin was included as a loading control. Canine mammary tissue was used as a positive control (+ Cont.). n = 3. Data are presented as mean ± standard deviation. (TIFF 336 kb) [file 12885_2018_4323_MOESM2_ESM.tif]
